# Supplementary material for: Splice-Junction-Based Mapping of Alternative Isoforms in the Human Proteome
Source: Cell Rep. Author manuscript; Available in PMC 2020 Jan 15. (PMC6961840; doi:10.1016/j.celrep.2019.11.026)
Supplement: 2 [file NIHMS1546469-supplement-2.zip › DF1/PXD006675/LeftAtrium/LeftAtrium_50_TPM1_AISEELDHALNDMTSGP.pdf]

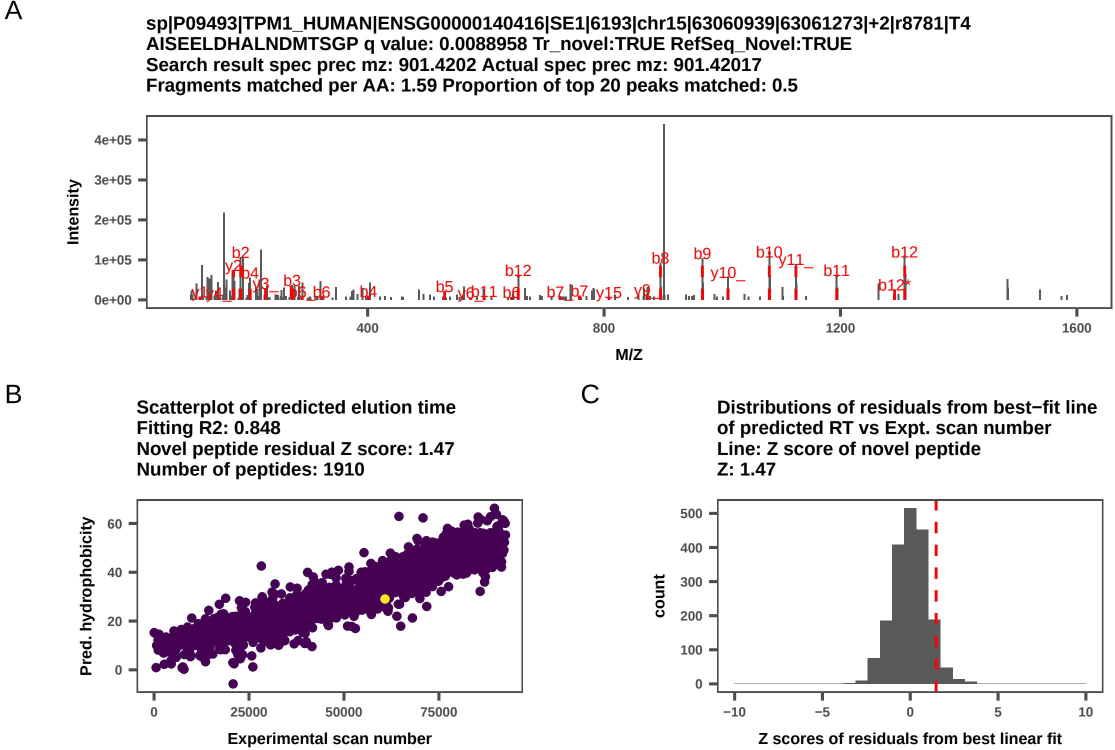
A
sp|P09493|TPM1_HUMAN|ENSG00000140416|SE1|6193|chr15|63060939|63061273|+2|r8781|T4
AISEELDHALNDMTSGP q value: 0.0088958 Tr_novel:TRUE RefSeq_Novel:TRUE
Search result spec prec mz: 901.4202 Actual spec prec mz: 901.42017
Fragments matched per AA: 1.59 Proportion of top 20 peaks matched: 0.5
Intensity
4e+05
3e+05
2e+05
1e+05
0e+00
M/Z
400
800
1200
1600
b2
b8
b9
b10
b12
b11
y10_
y11_
b12*
b5
b4
y15
B
Scatterplot of predicted elution time
Fitting R2: 0.848
Novel peptide residual Z score: 1.47
Number of peptides: 1910
Pred. hydrophobicity
60
40
20
0
Experimental scan number
0
25000
50000
75000
C
Distributions of residuals from best−fit line
of predicted RT vs Expt. scan number
Line: Z score of novel peptide
Z: 1.47
count
500
400
300
200
100
0
−10
−5
0
5
10
Z scores of residuals from best linear fit
